# Response to the Letter to the Editor Entitled “Desmopressin After Kidney Biopsy: Are the Risks Worth it?”

**DOI:** 10.1016/j.ekir.2025.07.010

**Published:** 2025-07-10

**Authors:** Narayan Prasad, Jeyakumar Meyyappan

**Affiliations:** 1 Department of Nephrology, Sanjay Gandhi Postgraduate Institute of Medical Sciences, Lucknow, Uttar Pradesh, India

The Author Replies:

We thank Cetin and Helvaci^1^ for their appraisal of our study^2^ in the letter to the editor “Desmopressin After Kidney Biopsy: Are the Risks Worth it?” and for acknowledging the robustness of our study’s design.

The major concern raised was the risk of patients experiencing side effects from desmopressin outweighing reduction in minor bleeding. Overall, the desmopressin group experienced a 21.4% lower risk of bleeding, 18.5% lower incidence of subcapsular hematoma, and 1.31 cm smaller hematoma size compared with the placebo group, with benefits persisting across all glomerular filtration rate groups. We agree that hematuria and hematoma are considered minor complications. Still, they impact patient outcomes, prolonging observation and hospitalization, necessitating repeated imaging and hemoglobin monitoring; enhancing anxiety to patients, care givers, and biopsy-performers; and posing significant clinical and logistical consequences in settings with limited access to interventional radiology or blood products. Unfortunately, our study could not demonstrate an impact on major bleeding requiring radiological intervention or nephrectomy, because such complications with real time ultrasound-guided kidney biopsy using an automated biopsy gun became rare and would require a larger sample size to draw a conclusion.

Desmopressin’s antidiuretic properties, causing transient water retention, resulted in hyponatremia and a decrease in hemoglobin, as an expected finding. We agree that the incidence of asymptomatic hyponatremia in the desmopressin group (∼29%) was high, similar to study by Rao and Chandra.^3^ We attributed this to higher intranasal dose (300 μg) in our study.^4^ Importantly, none of the cases were clinically symptomatic or required therapeutic intervention for hyponatremia. All patients with a sodium level < 130 mmol/l were excluded, suggesting a standard precaution if desmopressin is planned. Notably, hemoglobin decrease did not cause hemodynamic instability or require blood transfusions. Although worsening heart failure and volume overload are theoretical concerns, these were not observed in the present study, likely because most patients were sufficiently stabilized before the biopsy to tolerate lying supine during the observation period.

Our findings suggest using desmopressin before a kidney biopsy as a targeted, risk-adapted strategy to reduce the bleeding risk. The reductions in bleeding risk may outweigh the transient and manageable side effect profile, including headache and hyponatremia, as observed in our study.^2^ However, worsening of volume overload in anuric patients will remain a concern.
